# Mitochondrial hydrogen sulfide supplementation improves health in the *C. elegans* Duchenne muscular dystrophy model

**DOI:** 10.1073/pnas.2018342118

**Published:** 2021-02-24

**Authors:** Rebecca A. Ellwood, Jennifer E. Hewitt, Roberta Torregrossa, Ashleigh M. Philp, Justin P. Hardee, Samantha Hughes, David van de Klashorst, Nima Gharahdaghi, Taslim Anupom, Luke Slade, Colleen S. Deane, Michael Cooke, Timothy Etheridge, Mathew Piasecki, Adam Antebi, Gordon S. Lynch, Andrew Philp, Siva A. Vanapalli, Matthew Whiteman, Nathaniel J. Szewczyk

**Affiliations:** ^a^Medical Research Council (MRC) Versus Arthritis Centre for Musculoskeletal Ageing Research, Royal Derby Hospital, University of Nottingham, Derby DE22 3DT, United Kingdom;; ^b^Musculoskeletal Conditions, National Institute for Health Research Nottingham Biomedical Research Centre, Derby DE22 3DT, United Kingdom;; ^c^Department of Chemical Engineering, Texas Tech University, Lubbock, TX 79409;; ^d^Molecular Genetics of Ageing, Max Planck Institute for Biology of Ageing, 50931 Cologne, Germany;; ^e^Cologne Excellence Cluster on Cellular Stress Responses in Aging-Associated Diseases (CECAD), University of Cologne, 50931 Cologne, Germany;; ^f^University of Exeter Medical School, University of Exeter, EX1 2LU Exeter, United Kingdom;; ^g^Mitochondrial Metabolism and Ageing, Garvan Institute of Medical Research, Sydney, NSW 2010, Australia;; ^h^St. Vincent’s Clinical School, University of New South Wales (UNSW) Medicine, University of New South Wales Sydney, Sydney, NSW 2052, Australia;; ^i^Centre for Muscle Research, Department of Anatomy and Physiology, The University of Melbourne, Melbourne, VIC 3010, Australia;; ^j^HAN BioCentre, HAN University of Applied Sciences, Nijmegen 6525EM, The Netherlands;; ^k^Department of Electrical and Computer Engineering, Texas Tech University, Lubbock, TX 79409;; ^l^Sport and Health Sciences, University of Exeter, EX1 2LU Exeter, United Kingdom;; ^m^Living System Institute, University of Exeter, EX4 4QD Exeter, United Kingdom;; ^n^Ohio Musculoskeletal and Neurologic Institute, Ohio University, Athens, OH 45701;; ^o^Department of Biomedical Sciences, Heritage College of Osteopathic Medicine, Ohio University, Athens, OH 45701

**Keywords:** *C. elegans*, muscle, mitochondria, hydrogen sulfide, mouse

## Abstract

Duchenne muscular dystrophy (DMD) is an X-linked recessive disorder characterized by progressive muscle degeneration and weakness due to mutations in the dystrophin gene. The symptoms of DMD share similarities with those of accelerated aging. Recently, hydrogen sulfide (H_2_S) supplementation has been suggested to modulate the effects of age-related decline in muscle function, and metabolic H_2_S deficiencies have been implicated in affecting muscle mass in conditions such as phenylketonuria. We therefore evaluated the use of sodium GYY4137 (NaGYY), a H_2_S-releasing molecule, as a possible approach for DMD treatment. Using the *dys-1(eg*33*) Caenorhabditis elegans* DMD model, we found that NaGYY treatment (100 µM) improved movement, strength, gait, and muscle mitochondrial structure, similar to the gold-standard therapeutic treatment, prednisone (370 µM). The health improvements of either treatment required the action of the kinase JNK-1, the transcription factor SKN-1, and the NAD-dependent deacetylase SIR-2.1. The transcription factor DAF-16 was required for the health benefits of NaGYY treatment, but not prednisone treatment. AP39 (100 pM), a mitochondria-targeted H_2_S compound, also improved movement and strength in the *dys-1(eg*33*)* model, further implying that these improvements are mitochondria-based. Additionally, we found a decline in total sulfide and H_2_S-producing enzymes in dystrophin/utrophin knockout mice. Overall, our results suggest that H_2_S deficit may contribute to DMD pathology, and rectifying/overcoming the deficit with H_2_S delivery compounds has potential as a therapeutic approach to DMD treatment.

## Significance

Duchenne muscular dystrophy (DMD) is a fatal degenerative disease without a cure. Current standard pharmacological treatment is corticosteroids. Their prolonged use is associated with several undesirable side effects. Using *Caenorhabditis elegans*, we have identified pharmacological treatments that supplement hydrogen sulfide (H_2_S). One, sodium GYY4137, largely acts like prednisone to improve neuromuscular health; the other, AP39, targets H_2_S delivery to mitochondria. As these are not steroids, they are unlikely to produce steroid-induced side effects. Additionally, as DMD mice show a decline in total sulfide, our results pave the way for evaluation of cellular and/or mitochondrial H_2_S in DMD pathology and warrant further investigation of selective H_2_S delivery approaches in *mdx* mice and/or higher animal models of DMD.

Duchenne muscular dystrophy (DMD) is an X-linked neuromuscular disorder characterized by progressive muscle degeneration and weakness (1). It is caused by mutations in the gene that encodes for dystrophin, resulting in a decrease of functional dystrophin protein in muscle cells and neurons. Dystrophin links the intracellular cytoskeletal network to the transmembrane components of the dystrophin–glycoprotein complex, which provides stability to the muscle membrane. Destabilization of this complex from the reduction of dystrophin results in progressive muscle-fiber damage and membrane leakage (2). In addition, reduction of dystrophin causes increased sarcoplasmic calcium levels, which may cause a cascade of detrimental effects, including mitochondrial damage (3–7). DMD is a disorder that affects over 1 in 3,500 live male births. Prognosis is very poor, and currently there is no cure. Treatments are largely targeted at controlling the symptoms and focus on maximizing quality of life. Although the current standard pharmaceutical treatment, the corticosteroid prednisone, extends the ambulatory period by a couple of years, prednisone treatment is associated with several undesirable side effects, such as weight gain and behavioral difficulties, and may lead to osteoporosis (8). There has been an increased interest in exon-skipping therapies to make the condition resemble the less severe form Becker muscular dystrophy (9). Two antisense oligonucleotide-based therapies have received accelerated approval from the Food and Drug Administration, and two others are in clinical trials (10–12). However, this is still not a cure. Thus, there remains an unmet clinical need in seeking new and/or alternative treatments for DMD that can be used independently or in conjunction with other emerging therapies (13).

While the repurposing of medications approved for other uses in patients is a possibility, drug discovery often first utilizes animal and in vitro models to assess potential efficacy. DMD has been studied in mouse and canine models, as well as the invertebrate model *Caenorhabditis elegans* [reviewed by McGreevy et al. (14)]. *C. elegans* have a short lifespan, a low maintenance cost, a fully sequenced genome, and biological pathways conserved with humans (15). Due to its largely conserved musculature and neuromuscular junction, *C. elegans* has proven itself as a useful model for studying human muscle disorders, such as muscular dystrophy and sarcopenia (16–18). The DMD worm model has been employed for studying the use of pharmacological treatments and in a large-scale drug screen (19). Several models have been established for studying DMD by introducing mutations in *dys-1* (the dystrophin ortholog), which results in a loss of fully functional dystrophin. Here, we utilized *C. elegans* with a nonsense mutation at position 3,287 of the DYS-1 dystrophin ortholog (20). These *dys-1(eg*33*)* worms displayed impaired locomotion, reduced strength, elevated calcium levels, disrupted mitochondrial structure, and increased use of oxygen (5, 20–22). Importantly, the clinically used DMD drug prednisone has been shown to improve movement, strength, and mitochondrial structure in this model, confirming the utility of *dys-1(eg*33*)* worms as a DMD drug-screening platform (19, 22).

DMD and aging share some similarities, primarily the progressive loss of skeletal muscle mass (23). DMD is characterized by rapidly progressive muscle degeneration, and aging is usually associated with sarcopenia. *C. elegans* display a stereotypical histopathological progression of muscle decline both in DMD (24) and with age (25). In both cases, loss of mitochondrial structure is an early feature. Alterations in mitochondrial gene expression are known to occur prior to onset of symptoms in DMD *C. elegans* (26), and this impairment of mitochondrial function causes sarcomere disorganization (27). It has recently been shown that hydrogen sulfide (H_2_S), improved both survival and health of aging *C. elegans* by attenuating intracellular reactive oxygen species (ROS) generation and protecting against various stressors (28, 29).

H_2_S is a signaling “gasotransmitter” that is produced endogenously in mammals and in *C. elegans.* It is generated through the activity of three enzymes: cystathionine-γ-lyase (CSE), cystathionine-β-synthase (CBS), and 3-mercaptopyruavate transferase (3-MST). Endogenous H_2_S has been shown to have cytoprotective and antiapoptotic effects that may help to regulate various functions within the human body (28–30). In recent years, the use of H_2_S supplementation has been gaining attention due to its potential use in aging and age-associated diseases. H_2_S supplementation has been shown to slow the aging process by inhibiting oxidative stress and free-radical reactions [reviewed by Zhang et al. (30)]. Further to this, metabolic H_2_S deficiencies have been implicated in conditions such as phenylketonuria, which also affects muscle mass, highlighting the potential use of H_2_S supplementation in muscle-related disorders (31). We therefore hypothesized that H_2_S supplementation, using a slow-release H_2_S donor [sodium GYY4137 (NaGYY) (32)] and a mitochondria-targeted H_2_S delivery molecule [AP39 (33, 34)], would also give some beneficial effects in the *C. elegans dys-1(eg*33*)* model (22).

Here, we report that NaGYY improved DMD worm movement, strength, gait, and muscle mitochondrial structure. Similar to prednisone treatment, NaGYY treatment did not improve mitochondrial membrane potential. NaGYY also delayed muscle cell death, but did not significantly alter overall lifespan. The health improvements of both prednisone and NaGYY treatment required the action of the kinase JNK-1, the transcription factor SKN-1, and the NAD-dependent deacetylase SIR-2.1. The transcription factor DAF-16 was required for the health benefits of NaGYY treatment, but not prednisone treatment. Despite at least partially nonoverlapping mechanisms of action, combined treatment of DMD worms with both NaGYY and prednisone did not improve health more than either compound alone. The improvements seen are likely due to improving mitochondrial health, as NaGYY did not restore the normal calcium fluxes expected if dystrophin was restored and as confirmed using AP39 [the mitochondria-targeted H_2_S delivery molecule (33, 34)]. We also found a decline in total sulfide and in H_2_S-producing enzymes in a DMD rodent model, which suggests that there is a therapeutic utility with these compounds to overcome H_2_S deficiency in DMD skeletal muscle by targeting key intracellular components such as the mitochondria. Our results demonstrate the positive effects of H_2_S supplementation on DMD nematode health and open avenues of both therapeutic and mechanistic inquiry.

## Results

### NaGYY Treatment Improved *C. elegans* DMD Functional Defects in Movement, Strength, and Gait.

**Fig. 1.**
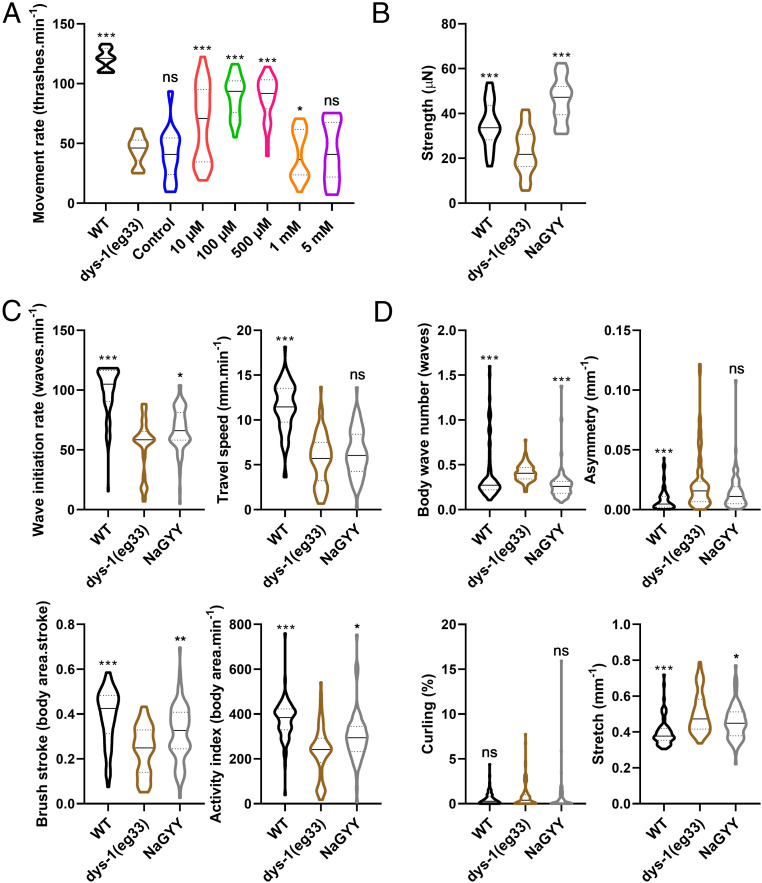
Effect of NaGYY on movement, strength and gait in *dys-1(eg*33*).* (*A*) *dys-1(eg*33*)* had a lower thrash rate than WT. NaGYY treatment significantly improved the thrash rate of *dys-1(eg*33*)* in a dose-dependent manner (10 µM to 1 mM). The NaGYY hydrolysis compound (100 µM), which was unable to generate H_2_S, was also assessed and showed no difference in thrash rate compared to *dys-1(eg*33*)*, demonstrating that it is H_2_S from NaGYY, and not the parent molecule or hydrolysis product formed after H_2_S generation, that was providing the beneficial effect. For all strains and treatments, *n* = 10 with five replicates with three biological repeats for a total of 150 data points per violin. Results were analyzed with a two-way ANOVA. All significance points are compared to *dys-1(eg*33*)*. *P < 0.05; ***P < 0.0001; ns, *P* > 0.05. (*B*) The *dys-1* mutant had reduced strength compared to WT animals. NaGYY(100 µM) improved the strength of *dys-1(eg*33*)* animals beyond WT levels. For all strains and treatment groups, *n* = 21 to 33. Results were analyzed with a one-way ANOVA. All significance points are compared to *dys-1(eg*33*).* ***P < 0.0001. (*C*) Activity parameters that decline with age also declined in the DMD worm model. NaGYY (100 µM) improved wave-initiation rate, brush stroke, and activity index, but not travel speed. (*D*) Morphological parameters which increase with age were also increased in the DMD model, with the exception of curling (no significant difference with respect to WT animals). NaGYY (100 µM) improved body-wave number and stretch, but did not improve asymmetry or curling. For all strains and treatments, *n* = 74 to 79. Results were analyzed by using a Kruskal–Wallis test. All significance points were compared to *dys-1(eg*33*).* *P < 0.05; **P < 0.01; ***P < 0.0001. ns, *P* > 0.05. Results are presented as a violin plot to show the frequency distribution of the data; the solid line represents the median, and the quartiles are represented by the dotted lines.

As is the case with DMD patients, worms with a mutation in dystrophin exhibit decreased movement (22). In worms, this can be assessed by measuring the worm’s thrash rate in a liquid medium (35). For all experiments, unless otherwise stated, L1 worms were synchronized to plates with or without compound and left to grow at 20 °C until day 1 of adulthood, when they could then be used. As shown in Fig. 1*A*, the decreased movement of *dys-1(eg*33*)* worms was attenuated by treatment with the slow-release H_2_S-generating molecule NaGYY. This improved movement was observed in a dose-dependent manner from 10 µM to 1 mM (Fig. 1*A*); 100 µM was subsequently used throughout this study unless otherwise stated. Control experiments performed by using hydrolyzed compound [synthesized as described in Alexander et al. (32)] incapable of generating H_2_S confirmed that the effects observed were due to H_2_S and not either the parent compound or hydrolysis product (Fig. 1*A*).

Recently, it has been shown that, as with DMD patients, *dys-1(eg*33*)* worms display muscle weakness (22). This report was enabled by a novel device called the NemaFlex (36), which has also been used to demonstrate muscle weakness in a worm-limb-girdle muscular dystrophy model (37). As shown in Fig. 1*B*, treatment with NaGYY also attenuated the strength loss observed in *dys-1(eg*33*)* worms.

Both DMD patients and *dys-1(eg*33*)* worms are known to display an altered gait, with patients displaying a waddling gait and worms displaying alterations in head and body bending (38–40). Recently, the *C. elegans* Swim Test (CeleST) has been developed as an objective method for assessing worm gait (41). CeleST reports eight different parameters, four of which are activity related, and the other four of which are morphological features. Activity-related parameters in *dys-1(eg*33*)* worms are shown in Fig. 1*C*; these include wave initiation rate, travel speed, brush stroke, and activity index (Fig. 1*C*). These activity-related parameters have been found to decline with age (41), and *dys-1(eg*33*)* also displays a decline in all these parameters compared with wild-type (WT). Aside from travel speed, all parameters were improved with NaGYY supplementation (Fig. 1*C*). Body wave number, asymmetry, curling, and stretch (Fig. 1*D*) are all morphological parameters that have been shown to increase with age (41). These four measures, apart from curling, are also increased in *dys-1(eg*33*)* compared to WT. NaGYY treatment reduced the number of body waves and improved stretch, but did not impact curling or asymmetry (Fig. 1*D*). These results suggested that DMD worms display gait abnormalities as seen in older worms and that NaGYY treatment improved most activity parameters, but largely did not improve the morphological features of the DMD worms’ altered gait.

### NaGYY Improved Mitochondrial Structure in *dys-1(eg*33*)* Worms.

**Fig. 2.**
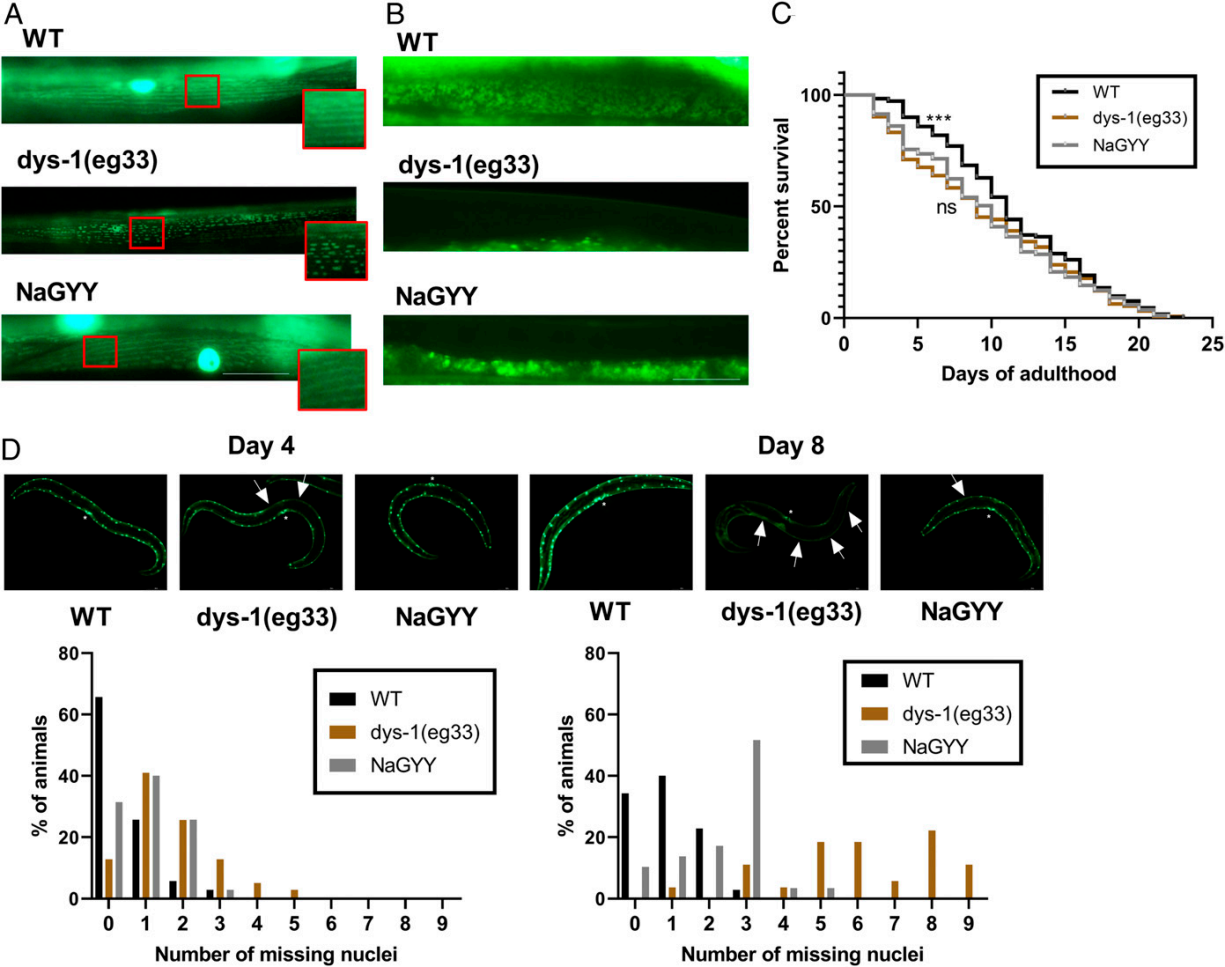
H_2_S ameliorated fragmentation of the mitochondrial network and delayed cell death, but did not improve defects in mitochondrial membrane potential or improve overall lifespan. (*A*) Representative images of CB5600 (WT with GFP-tagged mitochondria), which displayed a tubular mitochondrial network. CC91 [*dys-1(eg*33*)* with GFP-tagged mitochondria], which showed severe fragmentation of the mitochondrial network. NaGYY (100 µM)-treated CC91 animals displayed a normal mitochondrial network similar to that of WT. For all strains and treatments, *n* = 20. (*B*) JC-10 stained mitochondria in WT animals, and *dys-1(eg*33*)* showed a severely depolarized mitochondrial membrane, which is not improved by NaGYY (100 µM) treatment. For all strains and treatments, *n* = 20. (*C*) Lifespan curves were obtained by using the NemaLife. *dys-1(eg*33*)* has a shorter median lifespan (9 d) than WT (11 d), but NaGYY (100 µM) did not appear to extend lifespan in *dys-1(eg*33*)* animals (10 d). For all strains and treatments, *n* = 274 to 315, from two biologically independent repeats. Results were analyzed by using Kaplan–Meier curves, with Bonferroni-corrected multiple comparisons. ***P < 0.01. ns, *P* > 0.05. (*D*) Representative images of muscle-cell death in CB5600, CC91, and CC91 treated with NaGYY (100 µM). Muscle-cell death (as identified by the absence of muscle nuclei) increased with age in all animals, but was more severe in the *dys-1(eg*33*)* animals. NaGYY (100 µM) treatment appeared to slow the onset in cell death in *dys-1* animals. Vulva is identified by the *, and arrows show the missing nuclei. For all strains and treatments, *n* = 30. (Scale bar: 30 µm.)

Prednisone treatment has been shown to improve DMD worm movement and strength, and this was linked to improvements in mitochondrial structure (22). We therefore tested whether the beneficial effects induced by NaGYY shown in Fig. 1 were due to improved mitochondrial structure. As shown in Fig. 2*A*, *dys-1(eg*33*)* animals had a severely fragmented mitochondrial network that was improved by treatment with NaGYY. We therefore next examined the effects of NaGYY on mitochondrial membrane potential (ΔΨm) using the potentiometric fluorophore JC-10, which accumulates inside the mitochondrial membrane and exits the mitochondria based on mitochondrial membrane potential (35). Previously, prednisone had been shown to not significantly improve ΔΨm (22). This is also the case here, where NaGYY failed to improve ΔΨm, suggesting that H_2_S-mediated improvement of DMD worm health proceeds through similar mechanisms (i.e., mitochondrial structural improvement) as the clinically used drug prednisone (Fig. 2*B*). We then assessed overall lifespan of these animals and found that *dys-1(eg*33*)* (median 9 d, mean 9.5 d, and maximum 22 d) had a significantly reduced lifespan compared to WT (median 11 d, mean 11.5, and maximum 24 d). NaGYY did not significantly improve the lifespan of the *dys-1(eg*33*)* animals (median 10 d, mean 9.8 d, and maximum 21 d) (Fig. 2*C*). We also assessed muscle-cell death in these mutants with NaGYY treatment, as age-dependent muscle-cell death has been shown to be accelerated in *dys-1* mutants (20), and this acceleration was reduced with prednisone treatment (19). We found that NaGYY was able to delay muscle-cell death, suggesting an improvement in healthspan, but not in lifespan (Fig. 2*D*).

### NaGYY-Improved Movement Response Required the Same Genes in both Older Worms and DMD Worms.

**Fig. 3.**
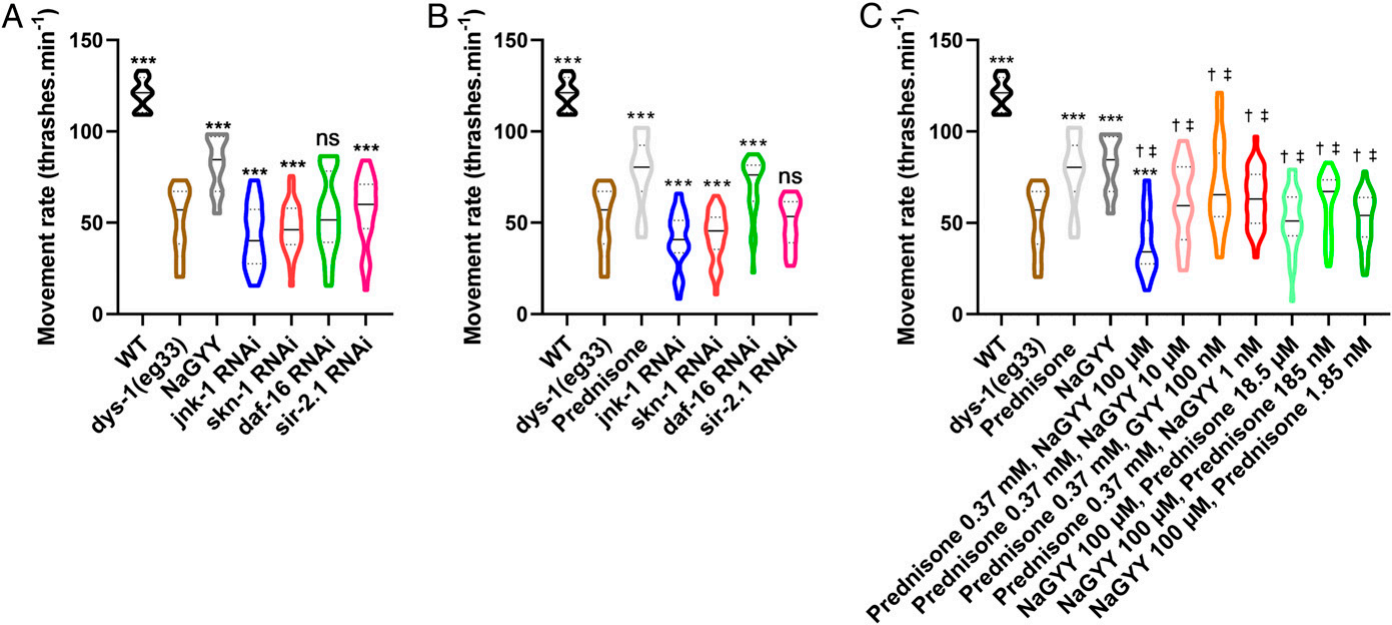
The mechanism of action of prednisone and NaGYY: The effect of RNAi treatment on aging- and stress-related genes and combined treatments. (*A*) NaGYY(100 µM) required the presence of *jnk-1*, *skn-1*, *daf-16*, and *sir-2.1* to improve movement in the *dys-1(eg*33*)* model. (*B*) Prednisone (370 µM) also required the presence of *jnk-1*, *skn-1*, and *sir-2.1* for its beneficial effect, but was able to largely restore movement in the absence of *daf-16*. (*C*) Combination therapy of prednisone and NaGYY did not provide increased beneficial effect. For all strains and conditions, *n* = 10 with five replicates and three biological independent repeats, equating to 150 points per bar. Results were analyzed with a two-way ANOVA. ***P < 0.001; ns, *P* > 0.05 [statistical significance compared to *dys-1(eg*33*)*]. ^†^*P* < 0.0001 (statistical significance compared to prednisone). ^‡^*P* < 0.0001 (statistical significance compared to NaGYY). Results are presented as a violin plot to show the frequency distribution of the data; the solid line represents the median, and the quartiles are represented by the dotted lines.

The mechanisms by which NaGYY treatment extended lifespan and improved healthspan in aging animals had been assessed previously by examining changes in gene expression in response to treatment (28, 29). Therefore, to determine if the mechanisms by which H_2_S improved health in DMD worms were largely the same as those in aging worms, we assessed the same genes in DMD worms. As shown in Fig. 3*A*, *jnk-1*, *skn-1*, *daf-16*, and *sir-2.1* were all required for the full beneficial effect of NaGYY treatment in DMD worms. While there was a small effect of NaGYY treatment in DMD worms with *sir-2.1* knocked down by RNA interference (RNAi), *sir-2.1* was clearly required for the full improved movement (Fig. 3*A*). These results suggested that the mechanisms by which NaGYY treatment improved health with age are likely the same mechanisms which improved health in DMD worms.

### Improved Movement in Response to Prednisone Treatment Did Not Require *daf-16*.

The molecular mechanism(s) responsible for prednisone’s clinical efficacy in DMD is still under investigation, but at least part of the mechanism is likely due to it its anti-inflammatory effects. The binding of prednisone (and other glucocorticoids) to the glucocorticoid receptor triggers the inhibition of the transcription factor nuclear factor kappa-light-chain-enhancer of activated B cells, which is responsible for causing inflammation [reviewed by Herbelet et al. (42)]. Although *C. elegans* lack an inflammatory system and satellite cells (16), prednisone is still able to improve DMD worm health. We therefore wanted to determine whether the mechanism(s) by which NaGYY treatment improved DMD health were similar to prednisone. As shown in Fig. 3*B*, *jnk-1*, *skn-1*, and *sir-2.1* are all required for the full beneficial effect of prednisone treatment on DMD worms. The set of genes required for the full beneficial effect of prednisone treatment is therefore similar to the results observed with NaGYY treatment on DMD worms (Fig. 3*A*). However, in contrast to treatment with NaGYY, prednisone did not appear to require *daf-16* for most of its beneficial effects. In the absence of *daf-16*, prednisone still resulted in a 30% increase in movement, compared to a 40% increase with *daf-16* present; this difference is not statistically significant (Fig. 3*B*).

These results suggest that the mechanisms by which NaGYY and prednisone treatment improve health in DMD worms are broadly similar, but that insulin signaling does not appear to be a major component of the mechanisms by which prednisone improves health in DMD worms.

### Combined NaGYY and Prednisone Treatment Does Not Provide Additional Health Improvement in DMD Worms.

There is increased evidence to suggest that future treatments in DMD will include combined therapies in order to optimize treatment efficacy (43). As NaGYY and prednisone appeared to have similar beneficial effects acting via at least partially distinct mechanisms, we next investigated whether utilizing a combined treatment of prednisone and NaGYY would provide a greater improvement in DMD worm movement than the treatments being given independently. As shown in Fig. 3*C*, when 370 µM prednisone and 100 µM NaGYY were given in combination, DMD worms experienced a decrement in movement. This result suggested an interaction between prednisone and NaGYY, perhaps due to overlapping mechanism(s) of action. To confirm that the interaction was reflective of the doses used in each treatment, we examined the dose dependence of the interaction. As shown in Fig. 3*C*, when either prednisone or NaGYY was used to treat DMD worms at the optimal dose (370 µM and 100 µM, respectively), and the other treatment was given in combination at lower doses, DMD worm movement improved, though not to the level when either compound was given independently. These results suggested that the interaction between the treatments was dose-dependent.

### NaGYY Given Postdevelopmentally Rapidly Improved Health.

**Fig. 4.**
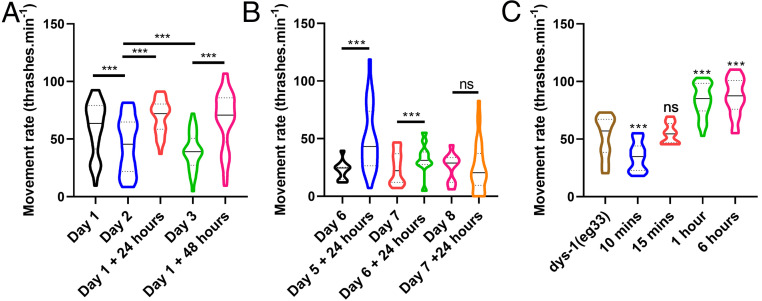
Acute treatment with NaGYY initially increased the movement capacity of *dys-1(eg*33*)* animals and delayed the age-related movement decline. (*A*) NaGYY (100 µM) improved movement both chronically and acutely. It initially increased the movement capacity of the animals, as shown after 24 h of exposure, and then it delayed the decline through the 48-h exposure. (*B*) NaGYY (100 µM) treatment given at day 6 of adulthood (postreproduction) still gave a beneficial effect 24 h after exposure. There was no improvement on day 7 adults after 24 h of treatment. (*C*) The effects of NaGYY (100 µM) was observed after 1-h exposure in day 1 adults. For all strains and conditions, *n* = 10 with five replicates and three biological independent repeats, equating to 150 points per bar. Results were analyzed with a two-way ANOVA. All significance points are compared to *dys-1(eg*33*).* ***P < 0.001. ns, *P* > 0.05. Results are presented as a violin plot to show the frequency distribution of the data; the solid line represents the median, and the quartiles are represented by the dotted lines.

Clinically, DMD patients are usually diagnosed before age 5, where diagnosis is defined by the onset of associated symptoms (44). Therefore, any potential treatment should have a beneficial effect during childhood and ideally during adulthood as well. Previously, chronic treatment with H_2_S has been shown to modulate the effects of age-related declines in muscle function, yet insulin-like signaling has been shown to act in adulthood to regulate *C. elegans* lifespan (45). Thus, we investigated whether NaGYY supplementation in adulthood could improve movement in adult DMD worms. As shown in Fig. 4*A*, DMD worms displayed a progressive decline in movement, as reported previously in aging WT worms (25). DMD worms were then grown to adulthood on noncompound plates. On day 1 of adulthood, worms were transferred to NaGYY plates to see if acute treatment would be useful. Acute treatment with NaGYY on day 1 of adulthood improved movement for at least the next 2 d (Fig. 4*A*). Acutely treating DMD worms with NaGYY can yield improved movement in animals as old as day 6 adults, as these animals exhibited higher movement 24 h after treatment. Day 7 adults treated with NaGYY for 24 h did not show an improved movement, suggesting this is the threshold at which no amelioration is observed (Fig. 4*B*). These results confirm that improved DMD worm health from NaGYY treatment can be achieved into the postreproductive stage for worms. As acute NaGYY treatment in adults gave an improved movement, we were curious as to how quickly movement could be improved in response to NaGYY treatment. As shown in Fig. 4*C*, movement improved within 1 h of treatment with NaGYY for day 1 adults (Fig. 4*C*), indicating the ability of NaGYY to rapidly improve animal movement.

### The Health Benefits of H_2_S Supplementation Appeared to Be Largely Mitochondria-Mediated.

**Fig. 5.**
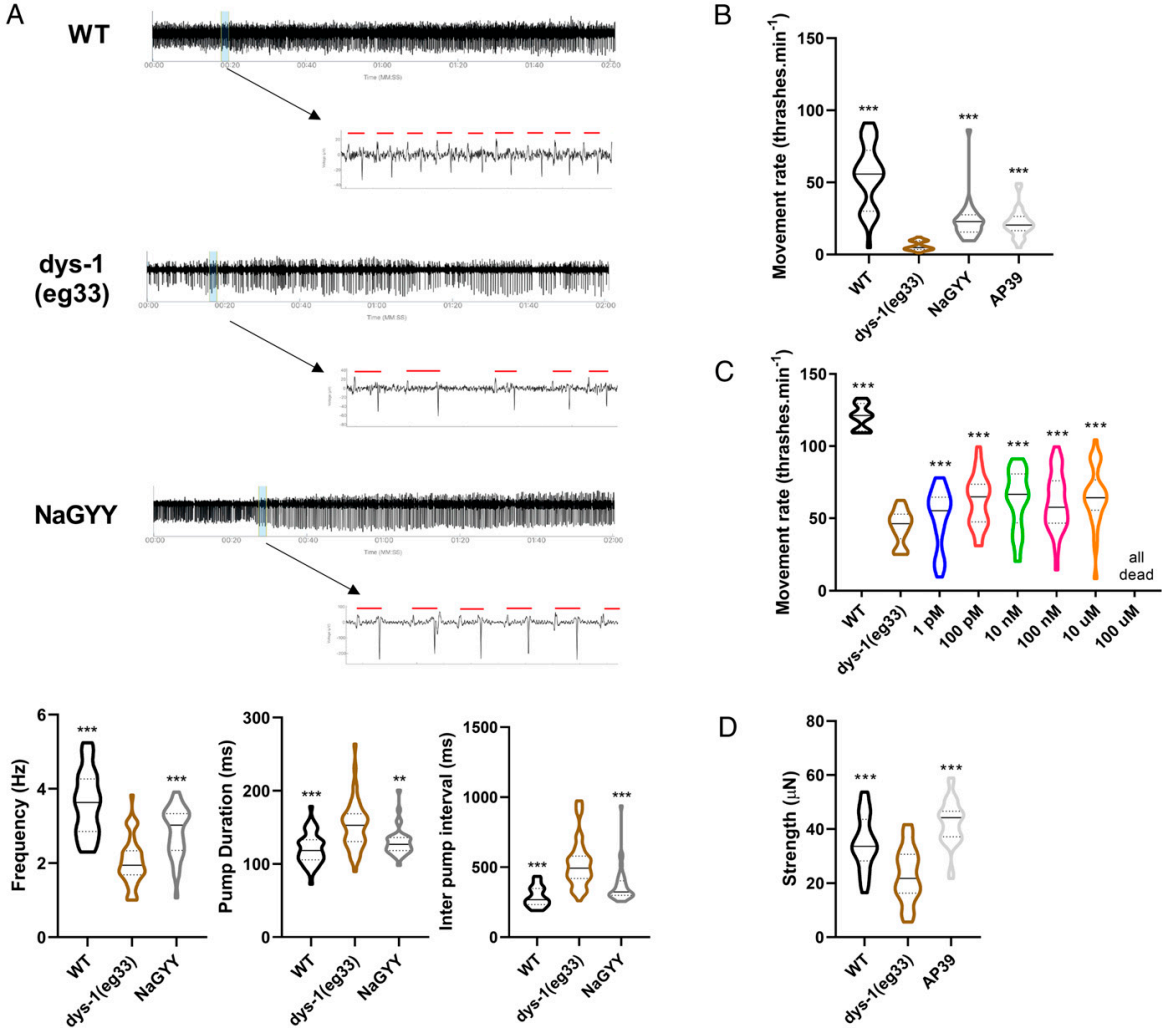
The physiological improvements from H_2_S supplementation were largely mitochondria-based. (*A*) EPG traces show a decline in pump frequency, an increase in pump duration, and an increase in the inter pump interval in *dys-1(eg*33*)* animals. NaGYY (100 µM)-treated animals showed a partial improvement in these parameters. For all strains and conditions, *n* = 15 with two independent biological repeats, equating to 30 data points per bar. Results were analyzed by one-way ANOVA. All significance points are compared to *dys-1(eg*33*).* **P < 0.01; ***P < 0.001. (*B*) *dys-1(eg*33*)* animals were hypersensitive to antimycin A. NaGYY (100 µM) and the mitochondria-targeted H_2_S AP39 (100 pM) ameliorated this hypersensitivity. (*C*) Dose–response curve of the thrash rate of *dys-1(eg*33*)* at different AP39 concentrations. (*D*) As with NaGYY (100 µM), AP39 (100 pM) treatment improved movement and strength of *dys-1(eg*33*)*-treated animals. For movement assay, all strains and conditions are *n* = 10 with five replicates and three biological independent repeats, equating to 150 points per bar. Results were analyzed with a two-way ANOVA. All significance points are compared to *dys-1(eg*33*).* ***P < 0.001. For strength, *n* = 21 to 33. Results were analyzed with a one-way ANOVA. All significance points are compared to *dys-1(eg*33*).* ***P < 0.0001. Results are presented as a violin plot to show the frequency distribution of the data; the solid line represents the median, and the quartiles are represented by the dotted lines.

A well-known underlying mechanism of DMD is loss of calcium homeostasis (3). We therefore assessed whether NaGYY was improving calcium handling. We used the NemaMetrix ScreenChip system to take electropharyngeogram (EPG) recordings of stimulated pumping, where each contraction is associated with an action potential (46). The traces for the *dys-1(eg*33*)* animals were significantly different from WT. They had a reduced frequency in pumping, an increase in pump duration, and an increased interpump interval (Fig. 5*A*). NaGYY treatment significantly improved these measures, but, like movement, not to WT levels. This observation suggested that, as with prednisone, restoration of dystrophin at the sarcolemma was unlikely the primary mechanism of action of NaGYY.

Given that *dys-1(eg*33*)* animals display reduced spare respiratory capacity (22), we hypothesized that they would be hypersensitive to mitochondrial electron-transport chain inhibition. The inhibitor antimycin A has been shown to cause paralysis and muscle-cell damage in WT animals due to the mitochondrial dysfunction (27). As shown in Fig. 5*B*, *dys-1(eg*33*)* animals were hypersensitive to treatment with antimycin A, and this hypersensitivity was significantly reduced by treatment with NaGYY. To demonstrate that H_2_S supplementation was reversing antimycin A hypersensitivity by acting directly in the mitochondria, we used the mitochondria-targeted H_2_S compound AP39 (33, 34). AP39 (100 pM) also reversed antimycin A hypersensitivity (Fig. 5*B*), confirming that specific intramitochondrial H_2_S supplementation was sufficient to rescue mitochondrial dysfunction in the DMD animals. We therefore confirmed that targeting H_2_S to the mitochondria using AP39 was also sufficient for improved physiological parameters of movement (dose–response in Fig. 5*C*) and strength (Fig. 5*D*).

### Sulfide Levels and H_2_S-Producing Enzyme Levels Were Reduced in Dystrophin/Utrophin Knockout Mice.

**Fig. 6.**
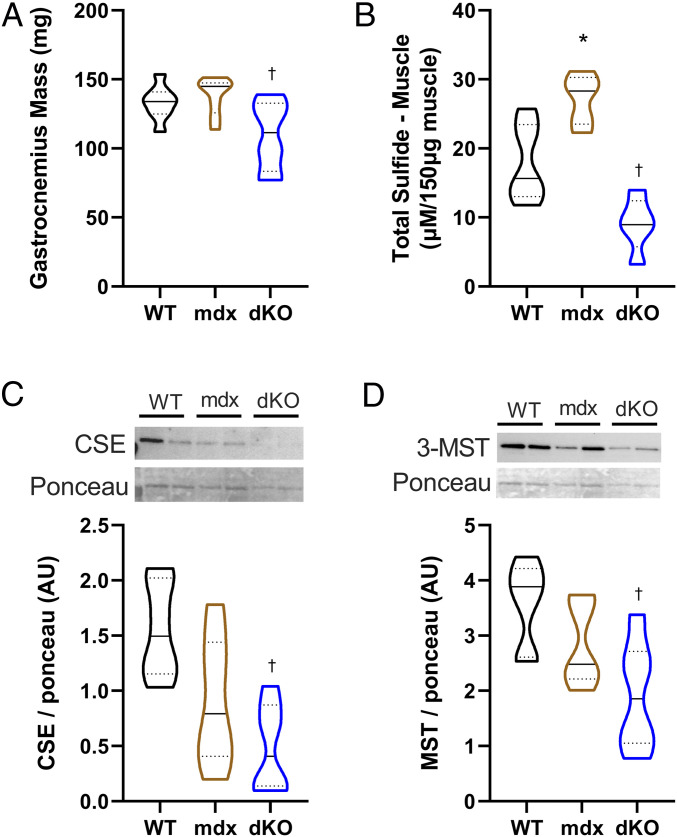
DMD rodent models displayed “H_2_S deficiency”: Total sulfide levels and expression of H_2_S enzymes were altered in DMD rodent models. (*A*) There was a decline in muscle weight in the dKO (utrophin/dystrophin deficient) mice compared to WT. (*B*) Total sulfide levels were increased in the *mdx* mouse and decreased in the dKO animals. (*C* and *D*) Protein expression of CSE and 3-MST were decreased in dKO animals, respectively. (*C* and *D*, *Upper*) Representative blots represent individual animals in each lane. *n* = 6 per condition. Results were analyzed with a one-way ANOVA. *P < 0.05 (significance for *mdx* relative to WT); ^†^*P* < 0.05 (significance for dKO relative to WT). Results are presented as a violin plot to show the frequency distribution of the data; the solid line represents the median, and the quartiles are represented by the dotted lines.

Finding that H_2_S supplementation improved DMD worm health raised the question of whether H_2_S levels, like NAD levels (47), were reduced across species in DMD. We therefore performed a pilot study in two rodent models of DMD alongside WT rodents (*n* = 6 per condition). We used the traditional *mdx* mouse model, which does not show any obvious clinical signs of the condition, and the more severe dystrophin and utrophin deficient double-knockout (dKO) mutant, which shows a closer resemblance to human DMD pathology (48). Consistent with past studies, we observed a decline in gastrocnemius muscle weight in the dKO animals, but not in the *mdx* mice (Fig. 6*A*). Total sulfide levels were significantly increased in the *mdx* animals and decreased in the dKO animals compared to WT (Fig. 6*B*). This suggests that the *mdx* animals may have a compensatory mechanism that is overwhelmed in the dKO animals. Thus, we examined the expression of the H_2_S-producing enzymes CSE and 3-MST. CSE is predominantly cytosolic and translocates to the mitochondria with stress, whereas 3-MST is mainly mitochondrial (49). Both enzymes were significantly decreased in the dKO animals (Fig. 6 *C* and *D*). Overall, these results imply that H_2_S metabolism is altered in DMD.

## Discussion

### Pharmacological H_2_S Improved Health in the *C. elegans* DMD Model.

H_2_S was previously considered a highly toxic gas. However, recent evidence demonstrated that it is endogenously produced in animals, which suggests that it may exhibit antiapoptotic, anti-inflammatory, and cytoprotective effects (50). H_2_S supplementation has been demonstrated to modulate the lifespan and healthspan of *C. elegans* (28, 29). Due to similarities in loss of muscle function with age and in DMD worms, we tested if H_2_S could improve DMD health. We determined that H_2_S administered in the form of the slow-release H_2_S donor NaGYY could improve DMD worm movement, gait, and strength, as well as improve muscle mitochondrial structure, all at a dose that was previously reported to improve *C. elegans* healthspan (28, 29). NaGYY, like prednisone (19), can delay muscle-cell death, but does not improve lifespan (Fig. 2 *C* and *D*). Notably, NaGYY can be administered postdevelopmentally and for several days postadulthood to improve worm movement, suggesting that H_2_S supplementation can be given acutely. A control compound has been used in this study, which provides further evidence that it is likely the H_2_S that has been supplemented that is having the therapeutic effect. A limitation of this study is that we did not know the drug concentration inside of the animal, only the dose that we administered. However, the dose we used was the same as what has been reported previously with commercial GYY4137 (28, 29). AP39, the mitochondria-targeted H_2_S compound, was also able to improve movement and strength in the *dys-1(eg*33*)* model. It is also important to note that AP39 was given at a substantially lower dose (100 pM) compared to NaGYY (100 µM) or prednisone (370 µM), meaning that toxicity is less likely. Although it is well known that DMD shows evidence of oxidative stress (51) and H_2_S has been proposed as an antioxidant (30), it is unlikely that the beneficial effects of NaGYY or AP39 were due to “oxidant scavenging” or direct “antioxidant” activity. Firstly, the rate constants of reaction of bolus sulfide with physiological oxidants such as O_2_^•−^, H_2_O_2_, ONOO− in vitro, even at concentrations several orders of magnitude higher than we have used (e.g., 5 mM NaSH), and in the absence of any competing biological milieu is far too slow to be physiologically relevant (52). Secondly, H_2_S generated via NaGYY (100 µM) (32) and AP39 (100 pM) (33, 34) is likely to slow, and resulting H_2_S levels would be insufficient even after complete hydrolysis to at least partially compensate for unfavorable reaction kinetics, even in the absence of competing cellular substates.

### Mechanisms Underlying Prednisone and NaGYY Treatment in Improved DMD Worm Health.

The primary cause of DMD is the loss of fully functional dystrophin protein, which results in a loss of membrane stability. In *C. elegans*, DYS-1 is required for the proper localization of several calcium channels (53–56). The mislocalization of these channels disrupts calcium homeostasis, resulting in elevated cytosolic calcium (5), a condition known to impair mitochondrial membrane potential [reviewed by Contreras et al. (57)]. Thus, the underlying loss of calcium homeostasis in DMD worms (5, 27) does not appear to be corrected by either prednisone or NaGYY treatment, since mitochondrial membrane potential is not restored by either treatment in DMD worms. EPG traces confirmed altered electrophysiologic function in DMD worms, and NaGYY treatment did not fully restore normal electrophysiologic function in DMD worms. Taken together, these results suggest that calcium homeostasis is improved, but not to an extent that allows for sustained improvements in mitochondrial membrane potential. Therefore, the calcium channels at the plasma membrane are likely still mislocalized, as DYS-1 levels are not restored in response to treatment with NaGYY.

However, the improved structure of muscle mitochondria (Fig. 2*A*) suggests that prednisone and NaGYY preserve mitochondrial integrity via an alternate mechanism. The ability of NaGYY coupled with the ability of AP39, the mitochondria-targeted H_2_S compound, to reverse electron-transport chain inhibition hypersensitivity, demonstrates that H_2_S is acting intramitochondrial in DMD worms. Furthermore, the ability of AP39 to restore movement and strength in DMD worms highlights that mitochondrial H_2_S supplementation is sufficient to improve DMD worm health. This raises the interesting question of whether prednisone and/or H_2_S supplementation is acting in a manner similar to NAD supplementation, which has also been shown to be beneficial in worm and rodent DMD muscle (47), or if they are acting in other ways to improve mitochondrial function to compensate for impaired membrane potential. The mitochondrial site of action likely explains the decreased cell death in response to NaGYY treatment. Importantly, it has recently been shown that while NAD supplementation does improve rodent DMD muscle, it does not fix all elements of the pathology (58). As H_2_S acts on mitochondria, we similarly do not anticipate that H_2_S supplementation will fix all elements of the pathology, a suggestion supported by the lack of lifespan improvement, despite cell-death improvement.

In addition to potentially having direct effects upon the respiratory chain (29, 59–61), prednisone and H_2_S could be improving health via activation of stress and/or adaptation pathways. Previously, *jnk-1*, *skn-1*, *daf-16*, and *sir-2.1* were shown to be required for the beneficial effects of H_2_S treatment on *C. elegans* lifespan extension (28). *jnk-1* is an ortholog of human MAPK, a stress-activated protein kinase (62), and has been shown to be a regulator of longevity and a mediator of the stress response (62, 63). Thus, as *jnk-1* was required for the full beneficial effects of both prednisone and NaGYY treatment, it appears that one mechanism of action of both treatments is via improved stress tolerance (Fig. 3 *A* and *B*).

*skn-1* and *daf-16* are both transcription factors and are orthologs of human NFE2 and FOXO, respectively. NFE2 is a regulator of oxidative stress and proteostasis (64), and FOXO is a regulator of metabolic and stress-responsive pathway-encoding genes [reviewed by Sun et al. (65)]. Thus, as *skn-1* was required for the full beneficial effects of both prednisone and NaGYY, both treatments appear to induce nuclear-encoded gene expression, which may have accounted for the improved mitochondrial structure observed in response to both treatments (Fig. 3 *A* and *B*). Similarly, as *daf-16* was required for the positive effects of NaGYY, but not prednisone, treatment, altered expression of metabolic genes may underlie part of the mechanisms of action of NaGYY, but not prednisone, treatment. This observation is interesting, given that prednisone can induce steroid diabetes with prolonged use (66, 67).

Lastly, *sir-2.1* is a human ortholog of sirtuin 1 (NAD-dependent deacetylase), which is involved in many cellular processes, including metabolism, stress, cellular senescence, and aging (68). Thus, there is a common theme: Genes required for the full therapeutic effects of prednisone and NaGYY are involved in the regulation of the stress response and mitochondrial gene expression. These observations seem consistent with the improved mitochondrial structure and overall animal health and suggest that DMD worms may be hypersensitive to any external stressors, such as the recently reported hypersensitivity to exercise (5).

### Future Directions.

Currently, there is no cure for DMD, and treatment options are limited. The main approved treatment is the use of glucocorticoid medications, with the most common form prescribed being the corticosteroid prednisone. Although the specific mechanism of prednisone is not fully known, long-term daily treatment has been shown to improve muscle strength and prolong independent ambulation from 6 mo to 2 y longer than in those not receiving corticosteroid (8). As with all steroid usage, there are often several undesirable side effects. In the short term, these are likely to be weight gain and mood changes, and in the long-term, side effects include growth suppression, thinning of bones, and diabetes (8). Our results suggest that not only should other compounds that have mitochondrial effects, such as prednisone, be examined for efficacy in treating DMD, but also that the combination therapy involving prednisone and decreased insulin signaling, could be examined. Furthermore, combination therapy with emerging exon-skipping and other treatments should also be explored.

The finding that NaGYY treatment recapitulated the beneficial effects of prednisone for DMD phenotypes in worms suggests that not only could H_2_S be examined as a potential treatment for DMD, but also for greater understanding of why H_2_S has therapeutic value and what the role of H_2_S is in the DMD pathology. AP39 treatment also improved movement and strength, suggesting that mitochondrial H_2_S has therapeutic value due to action in the mitochondria, such as improved cellular bioenergetics and adenosine triphosphate (ATP) synthesis, prevention of oxidant formation, preservation of mitochondrial DNA integrity, and delaying onset of cell death and inflammation (69, 70). Given that enzymes involved in the synthesis of H_2_S are known to translocate to the mitochondria in response to stress, H_2_S could potentially be produced in DMD muscle in order to compensate for mitochondrial stress. In particular, CBS and CSE translocate to the mitochondria in response to hypoxia to sustain ATP production (49). The 3-MST is found in both the cytosol and the mitochondria, but predominantly in the mitochondria. If this is the result of mitochondrial H_2_S depletion, then it is likely that the supplementation is acting to restore some mitochondrial function, much like NAD supplementation has been shown to do (47). The pilot data from DMD rodent models suggest that H_2_S levels are increased in *mdx* mice, but they are decreased in dKO mice. These data suggest that, H_2_S levels may increase in response to mitochondrial stress. Similarly, the pilot data from *mdx* and dKO mice suggest that H_2_S-producing enzymes may become depleted in response to increased mitochondrial stress, resulting in eventual failure of the H_2_S stress response. Further work is required to determine if these suppositions are true and if H_2_S supplementation in rodents is able to improve health. Similarly, if increased H_2_S improves mitochondrial function without altering membrane potential (as our data suggest in Fig. 2 *A* and *B*), the question arises of whether there are other treatments/mechanisms that could be similarly explored.

It is interesting to note that in both the worm and mouse models, animals are genetically identical [i.e., homozygous for the same mutation(s)], yet have variable individual results. It is unclear whether stochastic differences in global gene expression or environmental differences underlie the variability. Notably, both the response to H_2_S supplementation in worms and H_2_S-producing enzymes in rodents is highly variable at the individual level. This raises the interesting question of whether individual differences in mitochondrial function/homeostasis underlie individual differences in DMD severity.

Finally, we have shown that there are distinct changes in EPG traces in the worm DMD model. The pharynx is a muscular pump that contracts rhythmically during feeding and can be used as a model for the vertebrate heart (46). In ∼40% of cases, in DMD, the cause of death is cardiac failure, so the ability to study cardiac dysfunction in model organisms is invaluable (71). Our results raise the possibility of using the worm DMD model to gain greater mechanistic insight into DMD cardiac pathology, as well as screening for compounds to ameliorate it.

## Conclusion

This work demonstrates the beneficial effect of NaGYY on *dys-1(eg*33*)* health. Using clinically relevant phenotypes, we demonstrated the potential of H_2_S-releasing compounds in the treatment of DMD. AP39 alleviated the loss of muscle strength and ambulation in the *C. elegans* model, which is due in large part to improvements in mitochondrial integrity and function. Control experiments performed by using hydrolyzed compound (incapable of generating H_2_S) confirmed that the effects shown were due to H_2_S, and not either the parent compound or hydrolysis product. The mechanism of action of H_2_S requires further investigation, but we have shown that its mechanism overlaps with that of prednisone. Given that prednisone and NaGYY do not provide an enhanced effect when used in combination, identifying a different class of drugs with a different mechanism could also be beneficial. Overall, we provide evidence for the use of H_2_S compounds, including those which target H_2_S delivery to mitochondria, in the treatment of DMD and raise important questions of the role of H_2_S in the onset and progression of DMD pathology.

## Materials and Methods

### Strains and Culture Conditions.

*C. elegans* strains were cultured at 20 °C on Petri dishes containing nematode growth medium (NGM) agar and a lawn of *Escherichia coli* OP50, unless stated otherwise. Animals for the study were age-synchronized by gravity synchronization from the L1 stage and allowed to grow to the desired day of adulthood. The *C. elegans* strains used in this study were Bristol strain N2 (WT) and, *dys-1(eg*33*)* (strain BZ33), which has a nonsense mutation in the *dys-1* gene; both strains were provided by the *Caenorhabditis* Genetics Center. Mitochondrial network integrity was assessed by using CB5600 [ccIs4251 (Pmyo-3::Ngfp-lacZ; Pmyo-3::Mtgfp) I; him-8(e1489) IV] and CC91 [dys-1(eg33) I; ccIs4251 I; him-8(e1489) IV] (developed previously in this laboratory).

### Pharmacological Compounds.

Prednisone (Sigma-Aldrich) was dissolved in 100% ethanol and added directly to NGM after it had been autoclaved and cooled to 55 °C. The medium was immediately mixed and dispensed to 6-cm Petri dishes. The final concentration used was 370 µM, unless otherwise stated. This concentration was selected, as it falls within the range reported to reduce the number of degenerating cells in the *dys-1(cx18);hlh-1(cc561)* model and *dys-1(eg*33*)* model (19, 22). NaGYY and its inert hydrolysis product were synthesized as described by us (32). Commercially sourced GYY4137 is a morpholine salt (itself biologically active) complexed to undisclosed quantities of highly toxic, carcinogenic solvent [dichloromethane; xCHCl2; at least 1 dichloromethane:2 GYY4137 molecules (32)], which is metabolized by cells and in vivo to carbon monoxide (72, 73). We therefore used a pharmaceutically more acceptable sodium salt devoid of these two confounding factors, in addition to using its established and characterized hydrolysis product (32), to ensure that our observations were due to slowly released H_2_S, and not the parent molecule or hydrolysis product. Compounds were dissolved in double-distilled H_2_O (ddH_2_O) before being added to the surface of a seeded NGM 3-cm Petri dish ∼24 h before use. A thrash assay was used to determine the optimal dose of 100 µM for NaGYY, and this dose was used for all studies unless otherwise stated. AP39 was synthesized, as described by us (34). The compound was dissolved in dimethyl sulfoxide and diluted in ddH_2_O before being added to the surface of a seeded NGM 3-cm Petri dish ∼24 h before use. A thrash assay was used to determine the optimal dose of 100 pM (dose–response curve 1 pM to 100 µM was performed, and 100 pM was the lowest dose to give a beneficial effect), and this dose was used for all studies.

### Thrashing Assay.

Thrashing assays were carried out by picking a single adult animal into 20 μL of M9 buffer on a microscope slide. The number of bends in 10 s was counted and repeated five times for each worm for three independent biological replicates. These thrash counts were then multiplied by six to give the movement rate per minute. One body bend was recorded as one rightward body bend and leftward body bend. For each treatment, movement rates for 10 worms were measured with three biologically independent repeats (35).

### NemaFlex Strength Assay.

The NemaFlex is a microfluidic device containing an arena of pillars. The methods used to conduct the NemaFlex strength assay were as described (22, 36). However, a modified NemaFlex device was used so that worms could be assessed at an earlier stage of adulthood, as animals were day 2 adults for these Nemaflex experiments, but up to day 5 in the previous study (22). The distance from center to center of one pillar to another was 115 µM, and the pillar diameter was ∼44 µM on average; therefore, there was ∼71 µM of space that the worm could occupy between pillars. The pillar height was ∼87 µM. Worms were grown as described previously to day 2 adults. Briefly, the devices were loaded with one animal per chamber, and 1-min videos were taken at a rate of ∼5 images per second on a Zeiss Axio Observer 7 microscope with a 5x objective and a Hamamatsu OCRA-Flash4.0 digital camera. Movies were then processed by using in-house-built image-processing software (MATLAB, R2015b), where pillar deflections were converted to a force value. Approximately 30 worms were imaged per strain/treatment group.

### CeleST Swim Test.

CeleST [a specialized computer program that tracks the swimming behavior of multiple animals in liquid (41)] was used to explore swimming “gait” in WT, *dys-1(eg*33*)*, and drug-treated worms. Worms were grown as described previously to day 1 adults. Methods used were as described (74). Briefly, four or five animals were picked into the swimming zone (50 µL of M9 buffer into a 10-mm ring preprinted on a microscope slide), and 30-s movies were taken with ∼15 images per second on a LEICA MDG41 microscope at 1x magnification with a Leica DFC3000G camera. The analysis utilized a swim analysis program in MATLAB that read out on several parameters associated with aging. Approximately 60 worms were imaged per strain/treatment group.

### Mitochondrial and Cell Death Imaging.

Mitochondrial imaging was used in day 1 adults, with or without treatment, to examine the mitochondrial network. The CB5600 [ccIs4251 (Pmyo-3::Ngfp-lacZ; Pmyo-3::Mtgfp) I; him-8(e1489) IV] strain and CC91 [dys-1(eg33) I; ccIs4251 I; him-8(e1489) IV] were used for WT imaging and dystrophy imaging, respectively. Worms were cultured on NaGYY as described. Approximately 20 day 1 adults were picked into 20 µL of M9 buffer on a microscope slide with a coverslip applied. Worms were imaged at 40× magnification using a Nikon Eclipse 50i microscope. CB5600 and CC91 animals were also used for the cell-death images. The protocol used was as described (20). Briefly animals were assessed at day 4 and day 8 of adulthood, where the number of dead muscle cells was determined by quantifying the number of muscle cells that had lost their distinct circular nuclear GFP signal. Approximately 30 animals were picked into 20 µL of M9 buffer on a microscope slide with a coverslip applied. Worms were imaged at 10x magnification by using a Nikon Eclipse 50i microscope.

### JC-10.

JC-10 (Enzo Life Sciences, catalog no. 52305) was used to assess mitochondrial membrane potential. Worms used for assessing mitochondrial membrane potential were WT and *dys-1(eg*33*).* Approximately 40 day 1 adults were picked into 83 µM JC-10 in freeze-dried OP50 solution (LabTIE) for 4 h before imaging. Representative images were taken at 40× magnification by using a Nikon Eclipse 50i microscope.

### Lifespan Assay.

Lifespan assays were conducted by using the microfluidics-based Infinity Screening System (NemaLife Inc.). Worms were grown as described to day 0 of adulthood. Day 0 animals were then washed from plates with 2 mL of M9 and collected in conical tubes. Animals were washed three times with 14 mL of M9, allowing young adults to settle at the bottom, and the supernatant was removed to clear bacterial debris. Worms were then collected in a 2.5-mL sterile syringe, where ∼70 animals were loaded into each microfluidic chip (Infinity chip, NemaLife Inc.) for whole-life culture (75). Survival analysis began from day 1 of adulthood and was repeated daily until cessation of life. Each day, the microfluidic culture chips were washed to remove progeny and imaged for 90 s, followed by feeding of 20 mg/mL *E. coli OP50* in liquid NGM. Chips were then placed in Petri dishes with damp tissue wrapped in parafilm and stored at 20 °C until subsequent use. The acquired videos were scored for live/dead animals by using the Infinity Code software (NemaLife Inc.).

### Development of Animals on RNAi.

The RNAi feeding method was adopted in this study, where worms were fed bacteria expressing double-stranded RNA (dsRNA) (76). Starved L1s were synchronized onto standard RNAi plates with bacterial lawns expressing dsRNA for the relevant genes and allowed to reach day 1 of adulthood before assessment by thrash assay. Clones were obtained from the Open Biosystems Vidal Library. Clones used were as follows: *jnk-1*, B0478.1; *skn-1*, T19E7.2; and *daf-16*, R13H8.1. The *sir-2.1*: R11A8.4 clone was obtained from the Ahringer library. For each treatment, movement rates for 10 worms were measured with three biologically independent repeats.

### NemaMetrix ScreenChipTM EPG Recordings.

Synchronized worms were grown to L4 stage as described, with or without treatment. Worms were collected from the plates with M9 buffer and washed twice with M9 by centrifugation (2,500 rpm, 90 s). After washing, worms were resuspended in M9 containing 10 mM serotonin (5-hydroxytryptamine) and incubated for 10 min to stimulate pumping. EPG recordings were taken by using the NemaMetrix ScreenChip^TM^ system using an SC30 chip. Each EPG recording was 2 min in duration and analyzed by using the NemAquire software. Worms for each condition were analyzed in two independent experiments and the data combined, consisting of ∼30 animals. Representative images of the pumps were extracted from the NemAnalysis 0.2 software.

### Antimycin A Assay.

Worms were grown to day 1 of adulthood either with or without treatment as described. One milliliter of M9 buffer containing 2 µM antimycin A was added to a seeded NGM 3-cm Petri dish. Approximately 30 animals were picked into the buffer and left for 24 h at 20 °C. Individual animals were picked out of the buffer, and a thrash assay was carried out as described. For each treatment, movement rates for 10 worms were measured with three biologically independent repeats.

### Rodent Study.

#### Study approval.

Male, C57BL/10ScSn (BL/10), dystrophin-deficient (*mdx*), and dystrophin/utrophin-deficient (*dko*) mice were housed in the Biological Research Facility at The University of Melbourne under a 12-h light/dark cycle and provided access to drinking water and standard chow ad libitum. All experiments were approved by the Animal Ethics Committee (AEC 1212507 and 1613961) at The University of Melbourne and conducted in accordance with the Australian code of practice for the care and use of animals for scientific purposes as stipulated by the National Health and Medical Research Council (Australia). At the time of death, muscles and organs were excised, trimmed of nonmuscle tissue, blotted of excess moisture, weighed, and frozen in liquid nitrogen. Tissues were stored at −80 °C until further analysis.

#### Immunoblotting.

Gastrocnemius samples were powdered on dry ice by using a Cellcrusher tissue pulverizer (Cellcrusher Ltd.), with 25 mg of muscle homogenized via shaking in a FastPrep 24 5G (MP Biochemicals) at 6.0 m•s^−1^ for 80 s in a 10-fold mass of ice-cold sucrose lysis buffer (50 mM Tris, pH 7.5; 270 mM sucrose; 1 mM ethylenediaminetetraacetic acid (EDTA); 1 mM ethylene glycol tetraacetic acid; 1% Triton X-100; 50 mM sodium fluoride; 5 mM sodium pyrophosphate decahydrate; and 25 mM beta-glycerophosphate). Inhibitors were added fresh on the day of use and included 1 cOmplete protease inhibitor cocktail EDTA free tablet (Roche) and Phosphatase Inhibitor Cocktail 3 (Sigma-Aldrich). Samples were then centrifuged for 10 min at 8,000 × *g* at 4 °C to remove any insoluble material. Protein concentrations were determined by using the DC protein assay as per manufacturer’s instructions (Bio-Rad). An equal volume of protein (20 mg) was separated by sodium dodecyl sulfate/polyacrylamide gel electrophoresis on 12.5% gels at a constant current of 23 mA per gel for ∼60 min. Proteins were then transferred onto nitrocellulose membranes (Bio-Rad) by using a wet transfer system at 100 V for 1 h. Membranes were then stained in Ponceau S (Sigma-Aldrich) and imaged to check for even loading and transfer. Membranes were then blocked for 1 h in 3% dry milk in Tris-buffered saline with Tween (TBS-T). Membranes were incubated overnight in primary antibodies at 4 °C. Following primary antibody incubation, membranes were washed three times in TBS-T and subsequently incubated in the appropriate horseradish peroxidase-conjugated secondary antibody at room temperature for 1 h. Membranes were again washed three times in TBS-T prior to imaging. Images were captured by using the ChemiDoc (Bio-Rad) and quantified by using ImageJ.

#### Antibodies.

Polyclonal antibodies to 3-MST (ab85377) and CSE (ab151769) were from Abcam and used at a concentration of 1:1,000. Anti-rabbit secondary antibody (7074) was used at a concentration of 1:10,000 in TBS-T and was from Cell Signaling Technology.

#### Skeletal muscle sulfide analysis.

Total sulfide levels were determined by using the 7-azido-4-methylcoumarin (AzMC) fluorescent probe approach (Sigma-Aldrich, catalog no. L511455). Briefly, mouse skeletal muscle was powdered, and 50 mg was lysed using nondenaturing lysis buffer (50 mM Tris⋅HCl, pH 8.0; 150 mM NaCl; 1% Nonidet P-40; and 1% Triton X-100). The protein concentration was determined with DC Protein Assay (Bio-Rad). Tissue lysates (150 μg of total protein) were loaded into a black, opaque 96-well plate and combined with AzMC (0.01 mM). The plate was sealed and agitated at 500 rpm/37 °C for 60 min. Fluorescence of AzMC was measured by using a Clariostar Plus microplate reader (BMG LABTECH) at excitation λ = 365 and emission λ = 450 nm.

### Statistical Analysis.

All data are presented as violin plots from at least three replicates unless otherwise stated. Normality was first assessed by using the D’Agostino and Pearson tests, and then the statistical test was selected based on normality. Statistical differences were assessed by using either one-way ANOVA, two-way ANOVA, or Kruskal–Wallis test. Survival was analyzed by using Kaplan–Meier curves, with Bonferroni-corrected multiple comparisons. Significance was determined as *P* < 0.05, and all statistical analyses were completed by using GraphPad Prism.

## Data Availability

All study data are included in the article.
